# The association between leisure time physical activity in adolescence and poor mental health in early adulthood: a prospective cohort study

**DOI:** 10.1186/s12889-015-2658-5

**Published:** 2016-01-05

**Authors:** Per Hoegh Poulsen, Karin Biering, Johan Hviid Andersen

**Affiliations:** Danish Ramazzini Centre, Department of Occupational Medicine, University Research Clinic, Hospital West Jutland, Gl. Landevej 61, Herning, 7400 Denmark

**Keywords:** Adolescents, Gender, Leisure time physical activity, Depressive symptoms

## Abstract

**Background:**

The incidence of poor mental health (MH) is increasing in Denmark and worldwide, especially among 16–24 year olds. Low physical activity (PA) during adolescence seems to be a risk factor for poor MH in early adulthood. Among adults, it appears that a high level of PA may be protective against poor MH.

We aimed to examine whether high levels of leisure time physical activity (LTPA) during adolescence reduced the risk of poor MH at age 20/21.

**Methods:**

Prospective cohort study with data collected during 2004–2010 in the western part of Denmark. The study population was 3031 young people (age 14/15 in 2004). LTPA was the exposure variable and originates from questionnaires in 2004/2007. MH was the outcome variable and was measured at age 20/21 in 2010. MH was evaluated using a short version of the CES-DC. Logistic regression was used to analyse the associations between levels of LTPA and MH. All analyses were stratified by gender.

**Results:**

1,589 adolescents were included in the final analyses. Girls at 14/15 years of age with a low level of LTPA had an Adjusted Odds Ratio(AOR) of 1.63 (95 % CI = 1.23–2.17) for poor MH as 20/21 year olds, compared to girls with a high level of LTPA. Among boys, the corresponding AOR = 1.19 (95 % CI = 0.85–1.66). We found an exposure-response relationship between levels of LTPA and MH among girls, but not among boys. Girls with a reduction/persistent low level of LTPA between the ages of 15–18 had an increased risk for poor MH at age 20/21 compared to the reference group.

**Conclusions:**

Among girls, we found an association between a low level of LTPA among 14/15 year olds as well as a reduction/persistent low level of LTPA over time with poor MH at 20/21 years. We found no association between low levels of LTPA and poor MH among 14/15 year olds boys however it appears that a reduction/persistent low level of LTPA over time may have some influence on the risk of poor MH at 20/21 years. It is important to address the change in habits of LTPA during adolescence to prevent poor MH.

## Background

According to the World Health Organisation (WHO), depression is one of the largest burdens of disease in high-income countries, measured in disability adjusted life years and causing significant socio-economic costs [1, 2]. Worldwide, it is estimated that 350 million people are living with depression and globally the disease is the leading cause of disability among both women and men, with the highest incidence among women [3, 4]. Prevalence of depression among children and adolescents is estimated to be 20 % [5]. Among Danish adolescents, the prevalence of symptoms of mental health problems has been increasing from 2010 to 2013, where 8.2 % of young men and 17.5 % of young women aged 16–24 years reported mental health problems [6].

It is outlined in the literature that low socio-economic status (SES) defined by, for example, yearly household income and highest household education, female gender, high body mass index (BMI), lack of nuclear family in adolescence, smoking, alcohol and psychological health are among the most important potential risk factors for future mental health problems, as well as physical inactivity or a reduced level of physical activity (PA) during adolescence [7–13].

The Danish Health and Medicines Authority recommends that children and adolescents aged 5–17 years are physically active at least 60 mins a day (7 h/week). These recommendations are in line with international recommendations for PA by the WHO. The stated amount of PA is based on a qualified estimate based on evidence in the field to obtain a physically and mentally healthy life [14].

Some studies on PA have shown that boys tend to be more physically active than girls and studies indicate that girls more often stop exercising during adolescence [15–18]. Studies also show that half of boys and as many as two out of three girls do not meet the recommendations for PA and that this development has worsened over the years. There is a high drop-out rate during adolescence, which results in the fact that one in three 19 year olds does not exercise at all, which may be associated with an increased risk of future health problems [19].

### Physical activity and mental health

Intervention studies in adults suggest that PA may have an effect when treating depression, whereas a Cochrane review from 2013 comparing exercise with no treatment or standard treatment concluded that physical exercise had a modest clinical effect on depression [20]. The evidence of PA as a treatment for depression/depressive symptoms in children and adolescence is limited [21–24].

Some prospective studies in adults suggest that PA may have a protective effect against the development of poor mental health [25, 26], as does the recent systematic review by Mammen and Faulkner which included prospective studies (1988–2012) based on longitudinal studies on the association between PA and the prevention of depression in both children and adults. They found an inverse relationship between baseline PA and follow-up depression in 25 of 30 studies included in their review, suggesting that PA could be preventive in the onset of depression [27]. However, the results in children and adolescents are divergent. Some cross-sectional studies in the field show that a higher level of PA is associated with a lower level of depressive symptoms [12, 15, 28, 29]. Several prospective studies have examined the association between PA and the risk of future depression, where some studies show a weak association between a higher level of PA during adolescence and lower risk of future depression/symptoms of depression [9, 30] in boys [11, 31] and in girls [32], but others do not [10, 33, 34]. There is no consensus among the presented studies as to whether or not levels of PA may serve as a preventive factor in the development of future depression; however, there is a request for methodologically stronger studies that may increase the level of evidence in this area, because the existing literature mainly consists of cross-sectional studies and prospective studies with short follow-up periods.

The aim of this study is to examine whether a high level of leisure time physical activity (LTPA) during adolescence reduces the risk of future poor mental health as a 20/21 year old.

## Methods

### Design and population

The study is a prospective cohort study within an existing cohort.

Data were gathered as part of VestLiv, which is an ongoing survey following a complete regional cohort of young people born in 1989 and resident in the former county of Ringkoebing in the western part of Denmark. Project VestLiv was a longitudinal study of the relationship between socio-economic position and health in young people. The cohort was established in April 2004. The county had around 275,000 inhabitants. The project has so far included three waves of questionnaires (2004, 2007 and 2010) [35] which has been supplemented with a range of register-based information.

Participants were included in this particular study if they had responded to the question on LTPA in the first questionnaire round (14/15 years/2004). Detailed information on recruitment and data collection has been explained elsewhere [36].

**Fig. 1:**
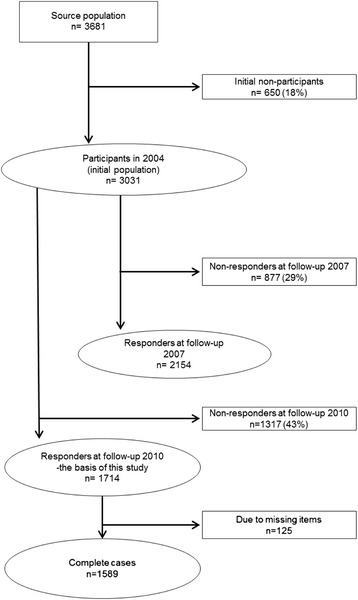
Distribution of participants and non-participants in 2004 and responders and non-responders (follow-up 2007 and 2010)

The study comprised 3681 young people (age 14/15), of whom 3031 responded to the question in the first questionnaire according to the weekly level of LTPA, resulting in an initial response rate of 82 %. Depending on the research question, attrition and missing data reduced the sample as shown in Fig. 1. This resulted in 1,589 participants for the final analysis in the study.

The following variables―mental health, LTPA and BMI— were collected from the questionnaires. Information on yearly household income and highest household education was derived from a national register in Statistics Denmark by using information from the Central Office of Civil Registration (CPR) in which the respondents are linked to their legal parents or guardians via a unique personal identification number given to everyone in Denmark at birth (or upon entry for immigrants).

### Variables

#### Definition of outcome

The primary outcome measure was mental health from the 2010 questionnaire where the participants were approximately 20/21 years of age.

Mental health was evaluated from an abbreviated version of the Center for Epidemiologic Studies Depression Scale for Children (CES-DC), which is a depression scale designed to measure current levels of depressive symptoms in a general population. It was originally developed for research purposes, but also used as a screening tool to identify persons at risk for clinical depression. The scale has been translated into several languages and validated for both young people and adults [37, 38]. It consisted of four items where the participant was asked about his or her mental state over the past week: "During the past week, how much have you had the following feelings?"

a. “I was happy this week”; b. “I felt like kids I knew were not friendly or that they didn´t want to be with me”; c. “I felt sad”; d. “It was hard to get started doing things this week”.

There were four categories of answer to each question in the form of not at all, a little, some and a lot.

The answers were subsequently awarded scores of 0–3, so that high values corresponded to having poor mental health. Response categories to question “a” were therefore reversed, and the answer category “not at all” given the score 3 and so on. The CES-DC scale was then calculated as a sum-score from 0–12. The definition of poor mental health was obtained by using the cutoff point of 3 and above as recommended for the shortened scale by Fendrich et al. [37]. Thus, mental health was in the project dichotomized as "good mental health" for people with score ≤ 2 and "poor mental health" for people with a score of ≥3.

Poor mental health and depression are two concepts that are closely related. Mental health problems are described by conditions such as depression and anxiety, as well as easier symptoms which for a shorter period may impede the person's quality of life and the ability to function and work [39]. Depression can be diagnosed by the expression of three core symptoms: decreased energy, increased fatigue and depressed mood, meaning sadness/depression, and loss of pleasure or interest in activities the person previously found pleasure in [39]. In this study the term “poor mental health” is used as a total concept that covers both depression and lighter depressive symptoms.

#### Definition of exposure

The main exposure variable was level of LTPA derived from the questionnaires in 2004 and 2007.

LTPA is a categorical variable with six possible answers, where each participant is asked in a single item, "How many hours a week do you usually exercise or play sports where you become breathless or have to sweat?”

The answer categories of LTPA were respectively: none, ½ h, 1 h, 2–3 h, 4–6 h and 7 h or more.

Level of LTPA is used as a dichotomous variable, a categorical exposure variable and a categorical variable of change.

#### Dichotomous exposure variable

“Low level of LTPA” (none, ½ h, 1 h and 2–3 h); “high level of LTPA” (4–6 h and 7 h or more).

#### Categorical exposure variable with three levels

“Low level of LTPA” (none, ½ h, 1 h and 2–3 h); “high level of LTPA” (4–6 h) and “very high level of LTPA” (7 h or more).

The selected cut-off values were based on studies by Holstein et al., McKercher et al., The Danish Health and Medicines Authority’s Health Profile, and The Danish Health and Medicines Authority’s health recommendations for PA for the age group [6, 9, 14, 18]. The cut-off for the high level of LTPA at approximately 4–6 h/week was chosen based on the fact that children and adolescents have approximately two weekly hours of PA at school [18] and thus close to the level of PA recommended by The Danish Health and Medicines Authority.

#### Change in level of LTPA (15–18 years):

**Fig. 2:**
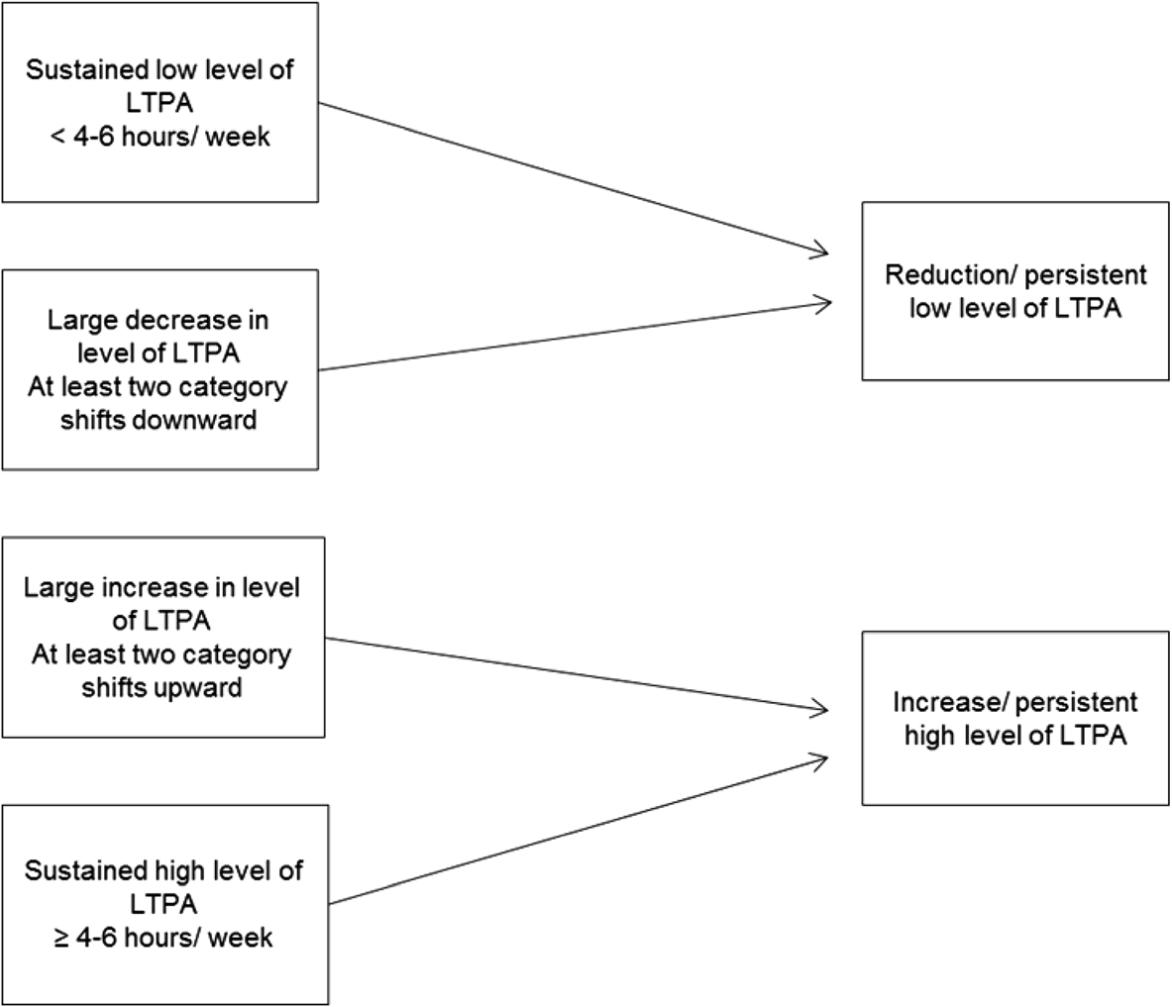
Change in levels of leisure time physical activity (2004/2007)

To deal with the change in levels of LTPA over time, the exposure variable is used as a difference defined as a category change between 2004 and 2007. This variable is categorized as, respectively, a large decrease in LTPA of at least two shifts downwards; sustained level of LTPA by switching to the neighbouring category or no change; and a great increase in level of LTPA by at least two shifts upwards. The variable is then dichotomized into “reduction/persistent low level of LTPA” and “increase/persistent high level of LTPA” respectively (Fig. 2).

### Potential confounders

BMI [11, 15, 32, 40], mental health (14/15 yr) [8–10, 41], yearly household income [15, 31, 41] and highest household education [17, 42] were chosen á priori as potential confounders. Smoking was tested as a potential confounder, but showed no association at all.

BMI was a continuous variable which was converted into a categorical variable with three categories. Participants were categorized as, respectively, “underweight” (BMI <17 kg/m^2^ for both girls and boys), “normal weight” (17 to 23.29 kg/m^2^ for boys and17 to 23.94 kg/m^2^ for girls) or “overweight” (BMI >23.29 kg/m^2^ for boys and BMI >23.94 kg/m^2^ for girls), using thresholds for 15 year old girls and boys [43]. The variable mental health (14/15 yr) was determined from the first survey, where the participants were asked to rate their mental health over the past week, as already described for the main outcome variable. Yearly household income was categorized, respectively, as low (<61.654 EUR), medium (61.654–80.381 EUR) or high (>80.381 EUR) income, grouped by 33.3^rd^ and 66.6^th^ percentiles [44]. Highest household education was divided into four categories: <10, 10–12, 13–15, >15 years.

### Statistical methods

Descriptive data of the analytic cohort are presented with complete cases overall and by gender. Comparisons were made for all the participants and non-participants between variables collected at the first questionnaire round and likewise for all the responders and non-responders at follow-up 2007/2010, respectively. The associations between outcome and exposure were calculated using logistic regression and reported as Odds Ratios (OR) with corresponding confidence intervals (95 % CI). The analyses were made with complete cases to ensure comparability between crude and adjusted estimates. To adjust for potential confounders— BMI and mental health (14/15 yr) —were included in the analyses as continuus variables, whereas yearly household income and highest household education were included as categorical variables.

We used Wald’s test for trend to examine the exposure-response relationship between levels of LTPA in 2004 and poor mental health at follow-up 2010.

All analyses were stratified by gender to examine if the association was different for girls and boys and to facilitate the comparison of results with other studies.

Data were analyzed using the statistical package Stata, statistical software version 13.1 (Stata Corporation, College Station, Texas, USA).

### Ethics

Use of the data is carried out under the same conditions and with the same purpose as when originally collected. Written consent was obtained from participants and their parents and data collection was reported to the Danish Data Protection Agency.

## Results

### Description of the study population

The analytic cohort consisted of 887 girls and 702 boys from the original cohort in 2004. As shown in Table 1, a larger proportion of boys than girls were physically active >7 h/week. Boys were more often overweight, had less often poor mental health at age 14/15 and a larger proportion of boys came from homes where highest household education was 13–15 years, compared to girls. 40 % of girls and 34 % of boys reported poor mental health at follow up 2010. Participants with poor mental health at age 20/21 had more often lower levels of LTPA, were more often overweight and came from homes with a lower yearly household income and highest household education.

**Table 1:** Characteristics of the analytic cohort at the first questionnaire round (2004) and in relation to poor mental health at follow up 2010 (N=1589)

	All	Girls	Boys	Poor mental health^a^
	n (%)	n (%)	n (%)	n (%)
Gender				
Girls	887 (56)			354 (40)
Boys	702 (44)			239 (34)
Variables–2004				
Physical activity				
Number of weekly hours				
None	20 (1)	12 (1)	8 (1)	8 (40)
1/2 hour	51 (3)	30 (3)	21 (3)	28 (55)
1 hour	161 (10)	92 (10)	69 (10)	61 (38)
2-3 hours	412 (26)	261 (30)	151 (21)	185 (45)
4-6 hours	575 (36)	324 (37)	251 (36)	195 (34)
7 hours or more	370 (24)	168 (19)	202 (29)	116 (31)
Body Mass Index				
Low (<17)	135 (9)	73 (8)	62 (9)	53 (39)
Normal (17/23.29^b^/23.94^c^)	1292 (81)	746 (84)	546 (78)	467 (36)
Overweight (> 23.29^b^/23.94^c^)	162 (10)	68 (8)	94 (13)	73 (45)
Mental health (14/15 yr)				
Good	1067 (67)	562 (63)	505 (72)	334 (31)
Poor	522 (33)	325 (37)	197 (28)	259 (50)
Yearly household income^d^				
Lowest	367 (23)	207 (23)	160 (23)	161 (44)
Middle	589 (37)	331 (37)	258 (37)	228 (39)
Highest	633 (40)	349 (40)	284 (40)	204 (32)
Highest household education				
<10 years	157 (10)	88 (10)	69 (10)	69 (44)
10-12 years	799 (50)	468 (53)	331 (47)	296 (37)
13-15 years	515 (32)	270 (30)	245 (35)	191 (37)
>15 years	118 (8)	61 (7)	57 (8)	37 (31)

^a^in relation to the All-category

^b^boys

^c^girls

^d^33.3rd;66.6th percentiles

When comparing all the participants to non-participants at the first questionnaire round, as well as comparing responders to non-responders at follow-up 2007/2010, we found boys more often than girls to be non-participants and non-responders at all three waves (Table 2). We also found that non-responders more often than responders had a lower level of LTPA and came from families where yearly household income and highest household education were lower. In addition, non-responders had a slightly higher proportion of poor mental health and overweight, compared to responders at follow up 2007/2010.

**Table 2:** Comparison of participants and non-participants at the first questionnaire round (2004) and responders and non-responders at follow-up 2007/2010 (*N*=3031)

	2004		Follow–up 2007		Follow–up 2010	
	Participants	Non-participants	Responders	Non-responders	Responders	Non-responders
	n (%)	n (%)	n (%)	n (%)	n (%)	n (%)
Gender						
Girls	1520 (50)	257 (40)	1164 (54)	356 (41)	961 (56)	559 (42)
Boys	1511 (50)	393 (60)	990 (46)	521 (59)	753 (44)	758 (58)
Variables – 2004						
Physical activity						
Number of weekly hours						
None	63 (2)	n/a	32 (1)	31 (3)	26 (2)	37 (3)
1/2 h	119 (4)	n/a	83 (4)	36 (4)	59 (3)	60 (5)
1 h	308 (10)	n/a	213 (10)	95 (11)	174 (10)	134 (10)
2–3 h	818 (27)	n/a	564 (26)	254 (29)	447 (26)	371 (28)
4–6 h	1023 (34)	n/a	752 (35)	271 (31)	618 (36)	405 (31)
7 h or more	700 (23)	n/a	510 (24)	190 (22)	390 (23)	310 (23)
Body Mass Index^e^						
Low (<17)	270 (9)	n/a	193 (9)	77 (9)	136 (9)	134 (11)
Normal (17/23.29^b^/23.94^c^)	2288 (80)	n/a	1653 (81)	635 (78)	1313 (81)	975 (78)
Overweight (>23.29^b^/23.94^c^)	302 (11)	n/a	197 (10)	105 (13)	165 (10)	137 (11)
Mental health (14/15 yr)^f^						
Good	1947 (65)	n/a	1394 (66)	553 (65)	1132 (67)	815 (64)
Poor	1030 (35)	n/a	731 (34)	299 (35)	565 (33)	465 (36)
Yearly household income^d^						
Lowest	889 (29)	336 (52)	541 (25)	348 (40)	411 (24)	478 (36)
Middle	1051 (35)	174 (27)	767 (36)	284 (32)	630 (37)	421 (32)
Highest	1090 (36)	138 (21)	845 (39)	245 (28)	673 (39)	417 (32)
Highest household education^g^						
<10 years	355 (12)	141 (23)	204 (10)	151 (18)	166 (10)	189 (15)
10–12 years	1537 (51)	330 (55)	1099 (52)	438 (51)	867 (51)	670 (52)
13–15 years	919 (31)	111 (18)	688 (32)	231 (27)	547 (32)	372 (29)
>15 years	168 (6)	21 (4)	136 (6)	32 (4)	121 (7)	47 (4)

^b^boys, ^c^girls

^d^33.3rd; 66.6th percentiles (1 missing), ^e^171 missings, ^f^54 missings, ^g^52 missings

### The association between levels of leisure time physical activity in adolescence and poor mental health at 20/21 years old

Among girls, we found that a low level of LTPA among 14/15 year olds and a reduction/persistent low level of LTPA over time were associated with poor mental health at 20/21 years (Table 3). Girls with a low level of LTPA had an Adjusted Odds Ratio (AOR) of 1.63 (95 % CI = 1.23–2.17) of having poor mental health as a 20/21 year old compared to the reference group. When applying the categorical exposure variable with three levels it appears that the association, between a very high level of PA and poor mental health, becomes even higher- with an AOR = 1.76 (95 % CI = 1.19–2.62). Wald´s test for trend was significant for girls (*p* < 0.001).

**Table 3:** The association between levels of leisure time PA during adolescence and poor mental health at age 20/21

	Girls (n=887)				Boys (n=702)			
	Crude OR	95 % CI	Adj. OR^h^	95 % CI	Crude OR	95 % CI	Adj. OR^h^	95 % CI
Dichotomous exposure variable								
High level of LTPA^i^	1		1		1		1	
Low level of LTPA	1.78	1.36–2.34	1.63	1.23–2.17	1.29	0.93–1.78	1.19	0.85–1.66
Categorical exposure variable with three levels								
Very high level of LTPA^i^	1		1		1		1	
High level of LTPA	1.12	0.75–1.66	1.12	0.75–1.69	1.12	0.75–1.66	1.08	0.72–1.62
Low level of LTPA	1.92^j^	1.31–2.80	1.76	1.19–2.62	1.37	0.92–2.03	1.24	0.83–1.87
Change in level of LTPA (15–18 yr)								
Increase/persistent high level of LTPA^i^	1		1		1		1	
Reduction/persistent low level of LTPA	1.69^k^	1.26–2.25	1.44	1.06–1.95	1.48^l^	1.05–2.09	1.36	0.95–1.93

^h^Adjusted for BMI, mental health (14/15 yr), yearly household income and highest household education

^i^Reference group

^j^ Wald´s test for trend (p<0.001), ^k^n=804, ^l^n=593

Girls also had a higher risk of poor mental health if they had a reduction/persistent low level of LTPA during adolescence (AOR = 1.44; 95 % CI = 1.06–1.95) compared to the reference group.

Among boys, there was no association between a low level of LTPA among 14/15 year olds and poor mental health however, when applying both the dichotomous and the categorical exposure variables, the estimates show a tendency that a low level of PA may increase the risk of poor mental health at age 20/21. Wald´s test for trend was non-significant in boys (*p* = 0.42).

The crude estimate for the association between a reduction/persistent low level of LTPA over time and poor mental health at 20/21 years was OR = 1.48 (95 % CI = 1.05–2.09). Adjustments for potential confounders however attenuated the association; AOR = 1.36 (95 % CI = 0.95–1.93).

## Discussion

### Main results

In this longitudinal study of 1,589 adolescents, we found that low levels of LTPA at the age of 14/15 were associated with poor mental health at the age of 20/21 in girls, but not in boys. Girls with a low level of LTPA at the age of 14/15 had a 60 % increased risk for poor mental health as a 20/21 year old, compared to girls who had a high level of LTPA. We also found an exposure-response relationship between levels of LTPA and poor mental health in girls.

Change in LTPA habits over time during adolescence to early adulthood appears to influence the mental health status at the age of 20/21, mainly in girls. Girls who had a persistent low level of LTPA or a reduction in levels of LTPA had a 40 % increased risk for poor mental health at the age of 20/21, compared to girls with an increase or a persistent high level of LTPA.

Boys had a 30 % increased risk for poor mental health if they had a persistent low or reduction in levels of LTPA during adolescence, compared to the reference group.

#### Comparison with existing literature

The gender difference in our study population with regard to being physically active as well as reporting poor mental health is in line with previous studies, which have reported that boys are more physically active than girls [15–18, 34], and that girls more often report their mental health to be poor [28, 33, 34, 45, 46].

We found consistency with previous longitudinal studies showing that low levels of PA in adolescence are associated with future poor mental health in girls [8, 32]. However, the studies by Sagatun et al. [31] and Flotnes et al. [11] suggest that the level of PA may have more influence on the development of mental health among boys than among girls. Sagatun et al. showed that weekly hours of PA (5–7 h) at age 15–16 years were weakly associated with mental health at the three year follow-up in boys, and the study by Flotnes et al. showed an exposure-response relationship with the number of days being physically active in relation to the risk of anxiety/depression symptoms in boys. We found an exposure-response relationship with weekly hours of LTPA and risk of poor mental health among girls, and the association among boys was in the same direction, eventhough not significant.

A recent longitudinal study by Toseeb et al. [34], who examined the association between exercise and depressive symptoms in adolescents (14/15 years old), could not support the hypothesis that PA protects against developing depressive symptoms. They hypothesized that those participants with higher levels of PA in early adolescence would have lower levels of depressive symptoms at the 2.5 year follow-up. In contrast, we found an association between a low level of LTPA in adolescence and a higher risk of poor mental health in early adulthood among girls. The reason for this difference in results between our study and the study by Toseeb et al. seems unclear; however, it may be attributed to different ways of measuring the level of PA, where Toseeb et al. used objectively measured PA and our study used self-reported levels of PA. Another explanation may be the different follow-up periods in the two studies, where we had six years of follow-up while Toseeb et al. had 2.5 years of follow-up.

Rothon et al. [10] examined the association between PA and depressive symptoms in adolescents. They found cross-sectional evidence that PA was inversely associated with depressive symptoms. However, they found no evidence for an association between a change in PA from baseline to follow-up and depressive symptoms at follow-up, although the direction of effect was the same as found in the cross-sectional analyses. We found an association between a change in LTPA habits and risk of poor mental health in girls. The two studies may show divergent results due to the different ages of the two studies’ populations, different cut-off values for levels of PA, shorter follow-up periods and different contexts.

The results from our study and the study by McKercher et al. [9] showed the same tendency, that a certain level of PA or an upward change in level of PA throughout adolescence may have a positive influence on the development of mental health in young adulthood.

Life in adolescence and into early adulthood is characterized by complexity and variability, both physiologically and mentally. There are shifts between schools, separations from parents, school work, increased demands from society regarding future plans, as well as an increase in risky behaviour regarding smoking and alcohol [17, 40, 47], all of which can affect the desire, as well as the ability, to be physically active in daily life.

### Strengths and limitations

#### Design and population

A methodological weakness of the study design was the fact that the exposure and outcome variable was based on self-reporting. This may result in information bias, which can lead to misclassification if participants systematically either over- or under-report their level of LTPA or mental health. Responders may wish to appear as healthy and active as possible, so responders may have a tendency to over-report their levels of LTPA. However, if the trend is the same for everyone, it would not affect the estimates. One might expect that responders with a low level of LTPA in particular may have over-reported their level of LTPA. Thus it could lead a non-differential misclassification of the exposure variable, which can cause bias towards the null hypothesis. Information on the outcome variable was also self-reported, but as responders should fill out scale questions about their own health, it seems unlikely that this would imply any misreporting. Additionally, it should be noted that responders were asked to note their mental health during the last week, so it seems unlikely that any bias should occur because of major recall problems.

650 (18 %) were non-participants for the cohort (Table 2). However, since the decision to participate in the original cohort was taken without the knowledge of any subsequent outcome in this study, we assume the case was non-differentiated selection on participation, which is not causing bias of the estimates.

The drop-out rate between 2004 and follow-up 2010 was 43 %. If drop-out was associated with both exposure and outcome, there would be a risk of differential non-response, which could bias the estimates. There were significant differences between responders and non-responders at follow-up in 2010. The initial levels of LTPA, as well as yearly household income and highest household education, were significantly different between the two groups―hence the possibility that there would be differences in the measured association. However, results from the study by Winding et al. [36], which used the same cohort, investigated whether non-participation and drop-outs influenced the size of the associations; it was found that neither non-participation nor drop-out had a significant influence on the size of the estimates, in spite of significant differences in, for example, SES [36].

One of the main strengths of our cohort was the prospectively collected data, with six years of follow-up. This design is suitable for observing changes over time and must therefore be considered to be appropriate for this kind of study. Another strength of the study is the measurement of the participants' habitual level of LTPA over time, which makes it possible to examine changes in the levels of LTPA and thus the influence that any change can have on a person's mental health during the transition from adolescence to early adulthood.

#### Measurement of mental health

Mental health was in this study dichotomized into good mental health and poor mental health, respectively, where the cut-off point was set at ≥3 as recommended by Fendrich et al.[37]. However, this cut-off is recommended for children and adolescents between the ages of 12–18, which might explain the rather high proportion of poor mental health among both girls and boys at the age of 20/21.

#### Measurement of levels of LTPA

Leisure time physical activity was analysed in both a dichotomized and categorized form. As a dichotomous exposure variable, LTPA was divided into low and high levels of LTPA, the cut-off point being <4–6 h/week for low levels of LTPA. When dichotomizing a categorical scale, information about the variable is lost [48]. LTPA used as a categorical exposure variable with three levels could have been used as a categorical exposure variable with four levels instead, to increase comparability with other studies, where 0 h/week would form the inactivity group, to examine the association between inactive adolescents and their risk of poor mental health, compared to adolescents with a very high level of LTPA. Hence we carried out an additional analysis with the categorical exposure variable with four levels; this, however, resulted in very wide confidence intervals due to there being few adolescents in the inactive group among both genders (data not shown).

#### Confounding

Adjustments were made for BMI, mental health (14/15 yr), yearly household income and highest household education.

One might have suspected that BMI could be part of the causal chain rather than a confounder, from the point of view that a low level of LTPA could lead to a higher BMI and thus lead to poor mental health. To investigate this assumption, we made an adjusted sub analysis without BMI and the size of the estimate was compared with that presented. For the girls, it increased the estimates slightly from 1.63 (95 % CI = 1.23–2.34) to 1.67 (95 % CI = 1.26–2.21) while for the boys, it increased the estimate slightly from 1.19 (95 % CI = 0.85–1.66) to 1.22 (95 % CI = 0.87–1.69).

#### Generalizability

The study setting is a predominantly rural area of Denmark, where the occupational structure is dominated by industry, commerce and agriculture. Analyses have shown, however, that the social structure is comparable to the rest of Denmark, although families in this part of the country are less likely to have a long educational background than in more urbanized areas [49]. Average disposable household income for members of the cohort is virtually identical when they are compared to peers of the same age from other parts of Denmark using register based information.

Therefore the results of the study may be transferred to young girls and boys with similar social and environmental conditions to this Danish population.

## Conclusion

This study found an association between levels of LTPA in adolescence and mental health in young adults among girls. The relationship among boys is tenuous. Change in the habits of LTPA levels during adolescence was associated with poor mental health at 20/21 years of age among girls and a tendency in the same direction was seen among boys.

### Perspectives/Implications of the research

It is important to address the change in habits of LTPA during adolescence to prevent future poor mental health, which seems to influence both genders in some way. It is of major concern that many adolescents are inactive or skip being physically active after the age of 15, because it may result in future health problems such as lifestyle diseases or mental health problems, all of which diminish quality of life and enhance societal costs. We know that good habits formed in childhood/ adolescence extend into adulthood and hence a bigger effort must be made to encourage and motivate adolescents to start being active or stay physically active. Since August 2014, the municipal primary schools in Denmark have implemented 45 mins of daily PA to enhance and improve the physical fitness of Danish children and adolescents, which it is hoped may help in preventing future health problems. This new implementation of daily PA in schools should be evaluated in future studies.

## Abbreviations

AOR: Adjusted Odds Ratio
BMI: Body mass index
CES-DC: Center for Epidemiologic Studies Depression Scale
CI: Confidence interval
EUR: Euro
LTPA: Leisure time physical activity
MH: Mental health
OR: Odds Ratio
PA: Physical activity
SES: Socio-economic status
Yr: Year

## References

[1] World Health Organization. Geneva 2008. The global burden of disease: 2004 update (data and statistics). http://www.who.int/healthinfo/global_burden_disease/GBD_report_2004update_full.pdf?ua=1. Accessed July 15, 2015.

[2] Danish Health and Medicines Authority. January, 2012. Depression. http://sundhedsstyrelsen.dk/da/sundhed/folkesygdomme/psykiatri/depression. Accessed July 15, 2015

[3] Vos T, Flaxman AD, Naghavi M, Lozano R, Michaud C, Ezzati M (2012). Years lived with disability (YLDs) for 1160 sequelae of 289 diseases and injuries 1990–2010: A systematic analysis for the Global Burden of Disease Study 2010. Lancet.

[4] Marcus M, Yasamy TM, Ommeren Mv, Chisholm D, Saxena S. 2012. Depression: A global public health concern. http://www.who.int/mental_health/management/depression/who_paper_depression_wfmh_2012.pdf?ua=1. Accessed July 15, 2015.

[5] Belfer ML (2008). Child and adolescent mental disorders: The magnitude of the problem across the globe. J Child Psychol Psychiatry.

[6] Christensen AI, Davidsen M, Ekholm O, Pedersen PV, Juel K. 2014. Danskernes Sundhed: Den Nationale Sundhedsprofil 2013. Danish Health and Medicines Authority

[7] Ammouri AA, Kaur H, Neuberger GB, Gajewski B, Choi WS (2007). Correlates of exercise participation in adolescents. Public Health Nurs.

[8] Jerstad SJ, Boutelle KN, Ness KK, Stice E (2010). Prospective reciprocal relations between physical activity and depression in female adolescents. J Consult Clin Psychol.

[9] McKercher C, Sanderson K, Schmidt MD, Otahal P, Patton GC, Dwyer T (2014). Physical activity patterns and risk of depression in young adulthood: A 20-year cohort study since childhood. Soc Psychiatry Psychiatr Epidemiol.

[10] Rothon C, Edwards P, Bhui K, Viner RM, Taylor S, Stansfeld SA (2010). Physical activity and depressive symptoms in adolescents: A prospective study. BMC Med.

[11] Flotnes IS, Nilsen TIL, Augestad LB (2011). Norwegian adolescents, physical activity and mental health: The Young-HUNT study. Nor Epidemiol.

[12] Moksnes UK, Moljord IEO, Espnes GA, Byrne DG (2010). Leisure time physical activity does not moderate the relationship between stress and psychological functioning in Norwegian adolescents. Mental Health Phy Activ.

[13] Ströhle A, Höfler M, Pfister H, Müller A, Hoyer J, Wittchen H (2007). Physical activity and prevalence and incidence of mental disorders in adolescents and young adults. Psychol Med.

[14] Pedersen BK, Andersen LB. 2011. Fysisk aktivitet- håndbog om forebyggelse og behandling. http://sundhedsstyrelsen.dk/publ/Publ2012/BOFO/FysiskAktivitet/FysiskAktivitetHaandbog.pdf. Accessed July 15, 2015.

[15] Bremnes AJ, Martinussen M, Laholt H, Bania EV, Kvernmo S (2011). The association between mental health and physical activity among high-school students. Tidsskrift for Norsk Psykologforening.

[16] Sund AM, Larsson B, Wichstrøm L (2011). Role of physical and sedentary activities in the development of depressive symptoms in early adolescence. Soc Psychiatry Psychiatr Epidemiol.

[17] Rangul V, Holmen TL, Bauman A, Bratberg GH, Kurtze N, Midthjell K (2011). Factors predicting changes in physical activity through adolescence: The Young-HUNT study. Norway J Adolescent Health.

[18] Holstein BE, Henriksen PE, Krolner R, Rasmussen M, Due P (2007). Trends in vigorous physical activity versus physical inactivity among 11–15 year olds from 1988 to 2002. Ugeskr Laeger.

[19] Ottosen MH, Andersen D, Nielsen LP, Lausten M, Stage S. 2010. Children and young people in Denmark- A national study on child well-being. Copenhagen.

[20] Cooney GM, Dwan K, Greig CA, Lawlor DA, Rimer J, Waugh FR (2013). Exercise for depression. The Cochrane Library.

[21] Larun L, Nordheim LV, Ekeland E, Hagen KB, Heian F (2006). Exercise in prevention and treatment of anxiety and depression among children and young people. Cochrane Database Syst Rev.

[22] Janssen I, le Blanc AG. Systematic review of the health benefits of physical activity and fitness in school-aged children and youth. The International Journal of Behavioral Nutrition and Physical Activity 2010; 7(40): Epub. 10.1186/1479-5868-7-40 PMC2885312 20459784

[23] Biddle SJ, Asare M (2011). Physical activity and mental health in children and adolescents: A review of reviews. Br J Sports Med.

[24] Bursnall P (2014). The relationship between physical activity and depressive symptoms in adolescents: A systematic review. Worldviews Evid Based Nurs.

[25] Mikkelsen SS, Tolstrup JS, Flachs EM, Mortensen EL, Schnohr P, Flensborg-Madsen T (2010). A cohort study of leisure time physical activity and depression. Prev Med.

[26] Jonsdottir IH, Rodjer L, Hadzibajramovic E, Borjesson M, Ahlborg G (2010). A prospective study of leisure-time physical activity and mental health in Swedish health care workers and social insurance officers. Prev Med.

[27] Mammen G, Faulkner G (2013). Physical activity and the prevention of depression: A systematic review of prospective studies. Am J Prev Med.

[28] Motl RW, Birnbaum AS, Kubik MY, Dishman RK (2004). Naturally occurring changes in physical activity are inversely related to depressive symptoms during early adolescence. Psychosom Med.

[29] Kremer P, Elshaug C, Leslie E, Toumbourou JW, Patton GC, Williams J (2013). Physical activity, leisure-time screen use and depression among children and young adolescents. J Sci Med Sport.

[30] Jacka FN, Pasco JA, Williams LJ, Leslie ER, Dodd S, Nicholson GC (2011). Lower levels of physical activity in childhood associated with adult depression. J Sci Med Sport.

[31] Sagatun A, Sogaard AJ, Bjertness E, Selmer R, Heyerdahl S (2007). The association between weekly hours of physical activity and mental health: a three-year follow-up study of 15-16-year-old students in the city of Oslo. Norway BMC Public Health.

[32] Neissaar I, Raudsepp L (2011). Changes in physical activity, self-efficacy and depressive symptoms in adolescent girls. Pediatr Exerc Sci.

[33] Hume C, Timperio A, Veitch J, Salmon J, Crawford D, Ball K (2011). Physical activity, sedentary behavior, and depressive symptoms among adolescents. J Phys Act Health.

[34] Toseeb U, Brage S, Corder K, Dunn VJ, Jones PB, Owens M (2014). Exercise and depressive symptoms in adolescents: A longitudinal cohort study. JAMA Pediatr.

[35] Danish Ramazzini Centre, Department of Occupational Medicine- University Research Clinic, Hospital West Jutland. VestLiv Project. http://www.vestliv.dk/dk/om-vestliv; http://www.vestliv.dk/dk/sprgeskema/tidligere-sprgeskemaer. Accessed July 15, 2015.

[36] Winding TN, Andersen JH, Labriola M, Nohr EA (2014). Initial non-participation and loss to follow-up in a Danish youth cohort: Implications for relative risk estimates. J Epidemiol Community Health.

[37] Fendrich M, Weissman MM, Warner V (1990). Screening for depressive disorder in children and adolescents: Validating the Center for Epidemiologic Studies Depression Scale for Children. Am J Epidemiol.

[38] Smarr KL, Keefer AL (2011). Measures of depression and depressive symptoms: Beck Depression Inventory-II (BDI-II), Center for Epidemiologic Studies Depression Scale (CES-D), Geriatric Depression Scale (GDS), Hospital Anxiety and Depression Scale (HADS), and Patient Health Questionnaire-9 (PHQ-9). Arthritis Care Res (Hoboken).

[39] Borg V, Nexø MA, Kolte IV, Andersen MF. Hvidbog om mentalt helbred, sygefravær og tilbagevenden til arbejde. http://www.arbejdsmiljoforskning.dk/da/publikationer/boeger-og-rapporter/boeger-og-rapporter?publicationId=597. Det Nationale forskningscenter for arbejdsmiljø 1. oplag, Kbh 2010.

[40] Wichstrøm L, von Soest T, Kvalem IL (2013). Predictors of growth and decline in leisure time physical activity from adolescence to adulthood. Health Psychol.

[41] Stavrakakis N, de Jonge P, Ormel J, Oldehinkel AJ (2012). Bidirectional prospective associations between physical activity and depressive symptoms, The TRAILS Study. J Adolesc Health.

[42] Brunet J, Sabiston CM, Chaiton M, Barnett TA, O'Loughlin E, Low NC (2013). The association between past and current physical activity and depressive symptoms in young adults: A 10-year prospective study. Ann Epidemiol.

[43] Cole TJ, Bellizzi MC, Flegal KM, Dietz WH (2000). Establishing a standard definition for child overweight and obesity worldwide: International survey. BMJ.

[44] Baadsgaard M, Quitzau J (2011). Danish registers on personal income and transfer payments. Scand J Public Health.

[45] Desha LN, Ziviani JM, Nicholson JM, Martin G, Darnell RE (2007). Physical activity and depressive symptoms in American adolescents. J Sport Exercise Psychol.

[46] Skrove M, Romundstad P, Indredavik MS (2013). Resilience, lifestyle and symptoms of anxiety and depression in adolescence: The Young-HUNT study. Soc Psychiatry Psychiatr Epidemiol.

[47] Helweg-Larsen K, Andersen S, Nielsen UB, Madsen M. Kønsperspektivet i unges trivsel og sundhedsadfærd i starten af det 21. århundrede. 2003.

[48] Juul S (2010). Epidemiologi og evidens.

[49] Glasscock DJ, Andersen JH, Labriola M, Rasmussen K, Hansen CD (2013). Can negative life events and coping style help explain socioeconomic differences in perceived stress among adolescents? A cross-sectional study based on the West Jutland cohort study. BMC Public Health.

